# Expression of trans-membrane serine protease 3 (TMPRSS3) in the human organ of Corti

**DOI:** 10.1007/s00441-018-2793-2

**Published:** 2018-02-19

**Authors:** Wei Liu, Hubert Löwenheim, Peter A. Santi, Rudolf Glueckert, Annelies Schrott-Fischer, Helge Rask-Andersen

**Affiliations:** 1 Department of Surgical Sciences, Section of Otolaryngology, Uppsala University Hospital, SE-751 85 Uppsala, Sweden; 2 Department of Otolaryngology, Head and Neck Surgery, Tübingen Hearing Research Centre, Eberhard Karls University Tübingen, 72076 Tübingen, Germany; 3 Department of Otolaryngology, University of Minnesota, 121 Lions Research Building, 2001 Sixth Street SE, Minneapolis, MN 55455 USA; 4 Department of Otolaryngology, Medical University of Innsbruck, Anichstrasse 35, A 6020 Innsbruck, Austria

**Keywords:** Cochlea, Trans-membrane Serine Protease 3 (TMPRSS3), Immunohistochemistry, Super-resolution structured illumination microscopy (SR-SIM), Human

## Abstract

TMPRSS3 (Trans-membrane Serine Protease 3) is a type II trans-membrane serine protease that has proteolytic activity essential for hearing. Mutations in the gene cause non-syndromic autosomal recessive deafness (DFNB8/10) in humans. Knowledge about its cellular distribution in the human inner ear may increase our understanding of its physiological role and involvement in deafness, ultimately leading to therapeutic interventions. In this study, we used super-resolution structured illumination microscopy for the first time together with transmission electron microscopy to localize the TMPRSS3 protein in the human organ of Corti. Archival human cochleae were dissected out during petroclival meningioma surgery. Microscopy with Zeiss LSM710 microscope achieved a lateral resolution of approximately 80 nm. TMPRSS3 was found to be associated with actin in both inner and outer hair cells. TMPRSS3 was located in cell surface-associated cytoskeletal bodies (surfoskelosomes) in inner and outer pillar cells and Deiters cells and in subcuticular organelles in outer hair cells. Our results suggest that TMPRSS3 proteolysis is linked to hair cell sterociliary mechanics and to the actin/microtubule networks that support cell motility and integrity.

**Electronic supplementary material:**

The online version of this article (10.1007/s00441-018-2793-2) contains supplementary material, which is available to authorized users.

## Introduction

TMPRSS3 (Trans-membrane Serine Protease 3) is a type II trans-membrane serine protease that belongs to a class of membrane-bound proteolytic enzymes whose genes are expressed in several tissues, including the fetal inner ear (Guipponi et al. 2002, 2008; Fasquelle et al. 2011). These membrane proteases are involved in diverse cellular activities and preferably interact with other surface proteins and with soluble proteins, matrix, and neighboring cells (Hooper et al. 2001). Their cytoplasmic domains also suggest that they have actions related to intracellular signal transduction (Wu 2003).

TMPRSS3 has been found in the developing cochlea, including the stria vascularis, spiral ganglion soma, modiolus, and organ of Corti (Guipponi et al. 2002). The TMPRSS3 gene encodes a protease containing a transmembrane domain, a low-density lipoprotein receptor class A (Südhof et al. 1985) that binds calcium (van Driel et al. 1987); a scavenger receptor cysteine-rich domain; and a C-terminal serine protease (Rawlings et al. 2010). According to Guipponi et al. (2008) and Fasquelle et al. (2011), TMPRSS3 is located and functionally active in the endoplasmic reticulum.

Several mutations in the TMPRSS3 gene on chromosome 21q22.3 cause autosomal recessive non-syndromic deafness (DFNB8/10) with varied phenotypic expression (Scott et al. 2001; Ben-Yosef et al. 2001; Lee et al. 2003; Guipponi et al. 2008). It is the first disorder in proteolytic activity found to result in hearing loss (Fasquelle et al. 2011). The mechanism of the pathology and deafness is still unclear. In a mouse mutant homozygous for TMPRSS3 (Y260X), Fasquelle et al. (2011) have detected the sudden degeneration of cochlear hair cells at the onset of hearing, suggesting its critical role in the maturation of the organ of Corti. TMPRSS3 was thought to regulate sodium homeostasis (Guipponi et al. 2002), but this is in doubt, since patients with ENaC gene mutations demonstrate normal hearing (Peters et al. 2006). Another proposed substrate is the potassium channel K_ca_1.1. In situ hybridization has shown that TMPRSS3 (Y260X) mRNA is localized in sensory hair cells in both the cochlea and vestibule, and patch-clamp and proteomic analyses have revealed that the outward K^+^ currents are altered in mutant mice. This has been confirmed by immunohistochemistry showing that the KCNMA1 channels, which are needed to generate outward-rectifying potassium currents, are absent at the neck of inner hair cells in TMPRSS3 (Y260X)-mutant mice (Molina et al. 2013).

Consequently, studies of the expression of TMPRSS3 are needed to improve our understanding of its biological role in the adult human inner ear and in ear disease. We have studied TMPRSS3 protein expression in the human cochlea with super-resolution for the first time via structured illumination fluorescent microscopy (SR-SIM) by using a polyclonal antibody developed against a recombinant protein hosted in the rabbit.

## Materials and methods

### Ethics statement

The study of human materials was approved by the local ethics committee (no. 99398, 22/9 1999, cont, 2003, Dnr. 2013/190 “Regionala Etikprövningsnämnden”), and subjects provided informed consent. The study adhered to the rules of the Declaration of Helsinki. Archival sections from adult cochleae were used (Liu et al. 2016). Pig and guinea pig cochleae were also analyzed in parallel. Ethical consent was obtained from the local ethical committee of Uppsala for animal use. The pig study protocol (permit number C108/4) and guinea pig study protocol (permit numbers C98/12 and C66/16) were approved by the Regional Animal Review Board of Uppsala, Sweden.

### Fixation and sectioning of human cochlea

Human cochleae from adult patients (Table 1) were collected from 2008 during trans-cochlear petro-clival surgery for life-threatening meningioma. In the operating room, the cochleae were dissected from the temporal bones and immediately placed in 4% paraformaldehyde diluted with 0.1 M phosphate-buffered saline (PBS; pH 7.4). After a 24-h fixation, the fixative was replaced with 0.1 M PBS and then with a 0.1 M Na-EDTA solution at pH 7.4 for decalcification. After 4 weeks, decalcified cochleae were rinsed with PBS. Before sectioning, cochleae remained immersed in 20% sucrose PBS overnight. After careful orientation of the cochlear specimens on the specimen holder (the axis of the modiolus was parallel to the surface of the specimen holder) in Tissue-Tek (OCT Polysciences), they were rapidly frozen on dry ice powder and cut into 8- to 10-μm sections by using a Leica cryostat microtome (CM1860, Leica Biosystem Nussloch, Nussloch, Deutschland). The frozen sections were collected onto gelatin/chrome-alum-coated slides and stored at −70 °C for immunohistochemistry.

**Table 1:** Patient data and method of analysis (*dB* decibels)

Age (years)	Sex	Pure tone threshold	Analysis
43	Female	50 dB (1–8 kHz)	Immunohistochemistry
51	Male	normal	
72	Male	50 dB (2–4 kHz)	
67	Female	Normal	
60	Male	Normal	Transmission electron microscopy
65	Male	Normal	

### Immunohistochemistry

The immunohistochemistry procedures used on the cochlear sections were as described in a previous publication (Liu et al. 2016). Briefly, sections were incubated on slides with a solution containing the antibodies in a humid atmosphere at 4 °C for 20 h. After being rinsed in PBS (3 × 5 min), the sections were incubated at room temperature for 2 h with a goat-derived secondary antibody (conjugated to either Alexa Fluor 488 or 555 (Sigma). The sections were counterstained with the nuclear dye DAPI (4′, 6-diamidino-2-phenylindole dihydrochloride) for 5 min and then rinsed with PBS (3 × 5 min). Sections were mounted with VECTASHIELD (for confocal microscopy) or ProLong Gold antifade reagent (Life Technologies, USA) mounting medium. Primary and secondary antibody controls and labeling controls were prepared to exclude endogenous labeling or reaction products. The sections used for the antibody control were incubated with 2% bovine serum albumin (BSA), omitting the primary antibodies. In the control experiment, no staining was visible of any structure of the cochleae. For TMPRSS3, we used a rabbit polyclonal antibody (Novus, #NBP1–85240, dilution 1:100). For K_Ca_1.1, we employed a rabbit polyclonal antibody (Alomone Labs, APC-107, dilution 1:100). Western blot analysis of rat brain membranes was performed with the two anti-K_Ca_1.1 antibodies (1184–1200, APC-107, 1:500 and #NBP1–85240, 1:500) pre-incubated with the control peptide antigen. For actin, we used MAB1501, which is a pan-actin antibody (Merck, monoclonal antibody from mouse, dilution 1:100) that binds to an epitope in a highly conserved region of actin; therefore, this antibody reacts with all six isoforms of vertebrate actin (Lessard 1988). The distribution of the six known isoforms of actin, namely four muscle actins (alpha-skeletal, alpha-vascular smooth, alpha-cardiac, and gamma-enteric smooth) and two cytoplasmic actins (alpha and gamma), is tissue-specific (Otey et al. 1986; Lessard 1988). The epitope recognized by the antibody is located in the N-terminal two-thirds of the actin molecule, near amino acids 50–70. It reacts with both globular (G) and filamentous (F) forms of actin and at a ratio as high as one antibody per two actin monomers; it does not interfere with actin polymerization to form filaments. However, this antibody does increase the extent of polymerization when used at a lower ratio of antibody to actin. In addition to labeling myotubes, anti-actin stains myoblasts and fibroblasts (Lessard 1988). Although clone C4 is prepared as an antibody against chicken gizzard muscle actin, it reacts with actins from all vertebrates and with *Dictyostelium discoideum* and *Physarum polycephalum* actins. Primary and secondary antibody controls and labeling controls were prepared to exclude endogenous labeling or reaction products (Burry 2011). Control sections were incubated with 2% BSA without the primary antibodies. The control slides showed no visible staining in cochlear tissues. Both wide-field and confocal fluorescence imaging software employed sensitive fluorescence saturation indicators to prevent overexposure. The specificity of the antibody was also demonstrated with Western blotting (Jia et al. 2016; www.novusbio.com/NBP1-85240). A mouse monoclonal antibody against parvalbumin (Millipore, #MAB1572, dilution: 1:300) was used to identify hair cells because, in humans, both outer hair cells (OHCs) and inner hair cells (IHCs) express parvalbumin (Table 2).

**Table 2:** Antibodies used in the study

Antibody	Type	Dilution	Host	Catalog number	Concentration	Company
Primary antibodies						
TMPRSS3	Polyclonal	1:100	Rabbit	NBP1–85240	0.05 mg/ml	Novus
K_Ca_1.1	Polyclonal	1:100	Rabbit	APC-107	0.6 mg/ml	Alomone Labs
K_Ca_1.1 (extracellular)	Polyclonal	1:50	Rabbit	APC-151	0.8 mg/ml	Alomone Labs
Pan-Actin	Monoclonal	1:100	Mouse	MAB1501	0.2 mg/ml	Millipore
Laminin β2	Monoclonal	1:100	Rat	# 05–206	1 mg/ml	Millipore
Secondary antibodies						
Anti-mouse IgG H&L; Alexa Fluor 555		1:400	goat	A21422	2 mg/1 ml	Invitrogen
Anti-rabbit IgG H&L; Alexa Fluor 488		1:400	goat	A11008	2 mg/1 ml	Invitrogen

### Imaging and photography

The stained sections were first investigated with an inverted fluorescence microscope (Nikon TE2000) equipped with a spot digital camera having three filters (for emission spectra maxima at 358, 461, and 555 nm). A computer system connected to the microscope was loaded with image-processing software (NIS Element BR-3.2; Nikon) that included an image merging capability and a fluorescence-intensity analyzer. For laser confocal microscopy, we used the same microscope equipped with a three-channel laser-emission system. The optical-scanning and image processing tasks were performed by using Nikon EZ-C1 (ver. 3.80) software, including the reconstruction of Z-stack images into projections and three-dimensional (3-D) images. Fluorescence intensity was quantified by area analysis of the proteins expressed in the organ of Corti by using the NIS Element BR-3.2 software. Super-resolution imaging was achieved with the SIM technique by using an inverted Zeiss LSM710 microscope equipped with the Elyra SIM module and 405-, 488-, 561-, and 633-nm lasers. The objective employed was a Plan-Apochromate 63×/1.4 oil DIC. The laser wavelengths for excitation were 405 nm (DAPI), 488 nm (Alexa 488), and 561 nm (Alexa 555). Emission was collected sequentially through bandpass filters: 420–480 nm for DAPI, 495–575 nm for Alexa 488, and 570–650 nm for Alexa 555. SIM processing was performed by using ZEN software at a variety of automatic settings, including the theoretical PSF, selection of noise filter setting, frequency weighting, and baseline settings, in order to evaluate the raw data. After evaluation, the SIM images were checked for possible artifacts, such as honeycomb patterns of intensity in the images so that appropriate selections of the evaluation settings could be applied. The optimal grid size was automatically assigned to each wavelength by the Zen software, and the grid was rotated 5 times at 5 phases for each image. Several optical sections were taken at various focal planes to create Z-stacks in the regions of interest. Then, the images were processed by using the default settings for the “structured illumination” function of the Zen software by transforming the 25 images that were acquired for each color at each focal plane into single high-resolution images. Both 2-D and 3-D striped-light patterns were used to illuminate the specimens. The microscope is capable of achieving a lateral (X-Y) resolution of approximately 100 nm and axial (Z) resolution of approximately 300 nm (Gustafsson et al. 2008). The resolution of the SIM system in BioVis (Uppsala) was measured with sub-resolution fluorescent beads (40 nm, Zeiss) in the green channel (BP 495–550 C LP750). An average PSF value was obtained from multiple beads with the built-in PSF experiment algorithm of the ZEN software. The typical resolution of the system was 107 nm in the X-Y plane and 394 nm in the Z plane (Liu et al. 2017b).

### Transmission electron microscopy

Specimens were collected both during surgery and after perilymph perfusion and were analyzed at the Ear Research Laboratories in Uppsala (*n* = 2; Rask-Andersen et al. 2000, 2012) and Innsbruck. The specimens were fixed in 3% phosphate-buffered glutaraldehyde and rinsed in cacodylate buffer, followed by post-fixation with 1% osmium tetroxide at 4 °C for 4 h. Decalcification was performed in 0.1 M Na-EDTA for 4 weeks. The specimens were infiltrated with Epon resin in a vacuum chamber for 4 h. For analysis by transmission electron microscopy (TEM), the sections were viewed with a JEOL 100 SX electron microscope (Uppsala) or a Zeiss LIBRA 120 electron microscope (Institute of Zoology, Innsbruck). For TEM, the resolution point-point was 0.34 nm, and the information limit was <0.20 nm in the x–y direction (Zeiss Libra 120). The z-resolution was limited by section thickness, which was chosen to be 80–90 nm to achieve pertinent contrast.

## Results

**Fig. 1:**
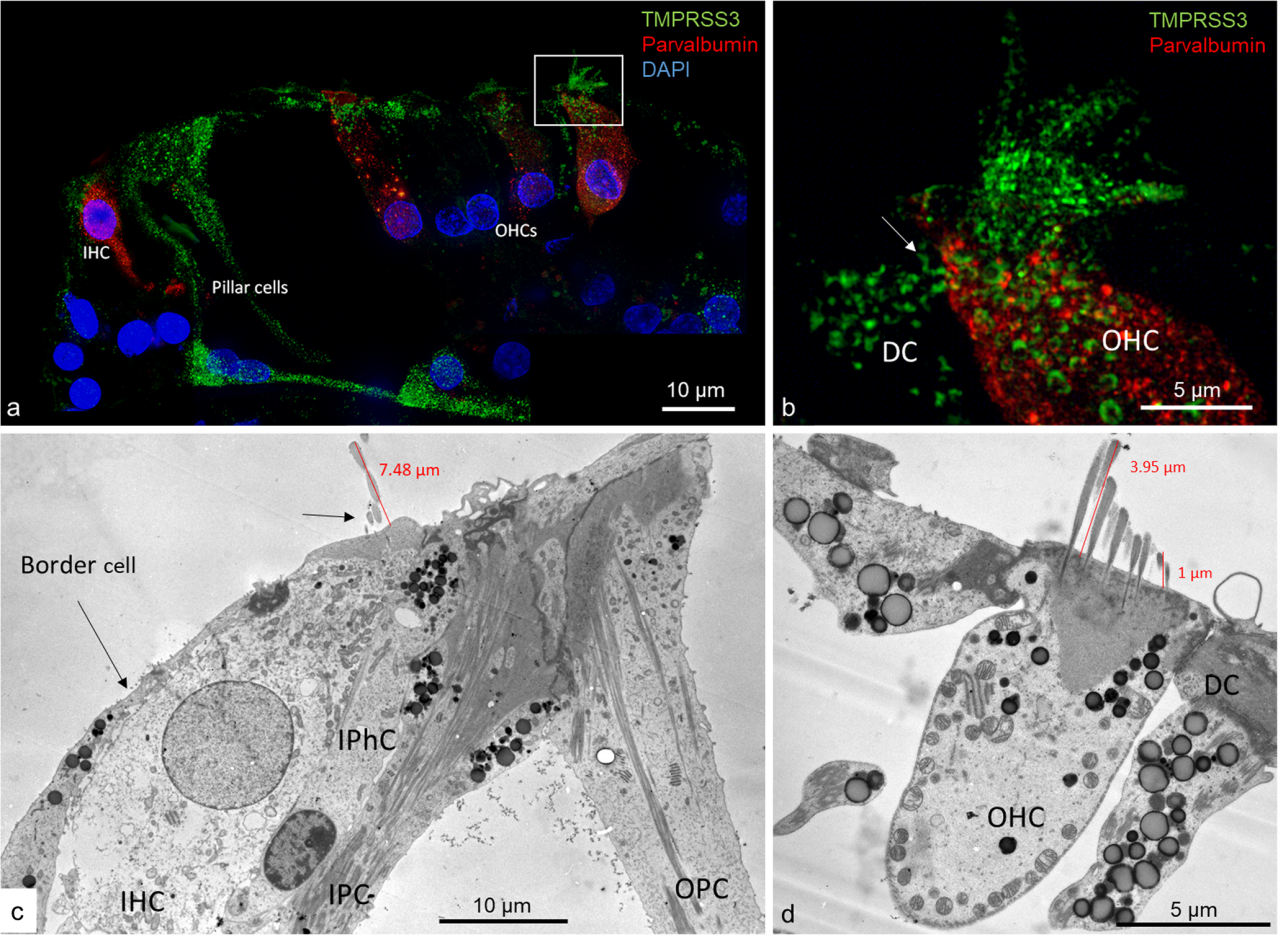
Super-resolution structured illumination microscopy (SR-SIM) of parvalbumin/TMPRSS3 immunohistochemistry in the human organ of Corti and transmission electron microscopy (TEM) at the corresponding region. **a** SR-SIM image (maximum intensity projection) of parvalbumin/TMPRSS3 immunoreactivity in the human organ of Corti. TMPRSS3 (*green*) is strongly expressed in hair cell stereocilia and in supporting cells. Pillar cells show extensive protease activity, particularly at the head and foot regions. The OHC cytoplasm contains vesicles expressing TMPRSS3. *Framed area* in **a** is shown at higher magnification in **b**. Nuclei appear in *blue* (*DAPI* 4′, 6-diamidino-2-phenylindole dihydrochloride). **b** TMPRSS3 staining can be seen in Deiters cells (*DC*, *arrow*) and in outer hair cells (*OHC*). Both OHCs and inner hair cells (*IHC*) express parvalbumin (**a**, **b**). **c**, **d** TEM of a human IHC and OHC at corresponding regions. The IHC contains no vesicles (*IPhC* inner phalangeal cell, *IPC* inner pillar cell, *OPC* outer pillar cell)

**Fig. 2:**
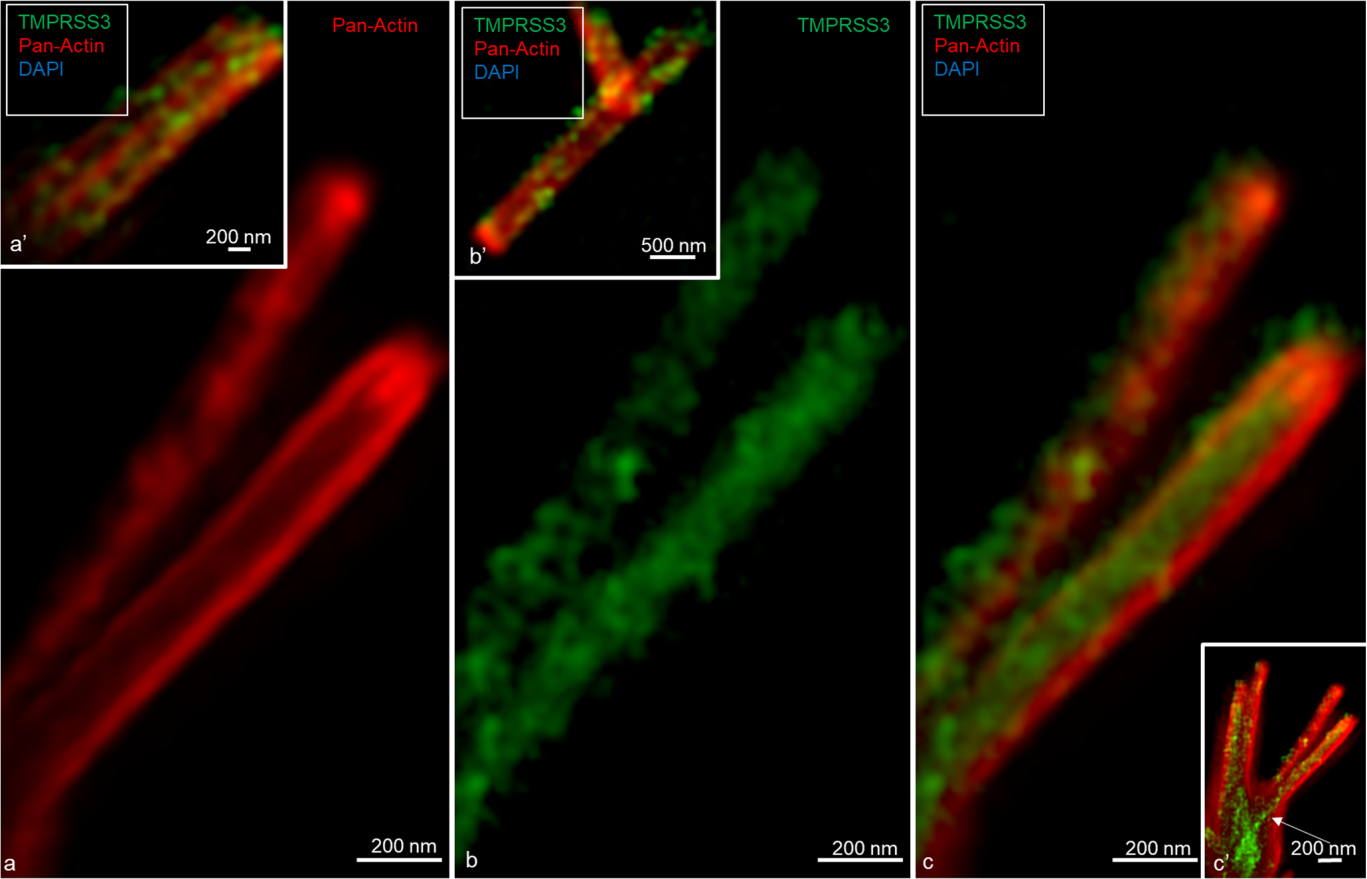
SR-SIM (maximum intensity projection) of human IHC stereocilia immunohistochemically co-labeled with antibodies against actin and TMPRSS3. **a** Immunohistochemistry showing presence of actin in the stereocilia. **b** Immunohistochemistry showing presence of TMPRSS3 in the stereocilia. **c** Merged images **a** and **b**. **a’**, **b’** OHC stereocilia. **c’** IHC stereocilia and the cuticula (*arrow*) at lower magnification

**Fig. 3:**
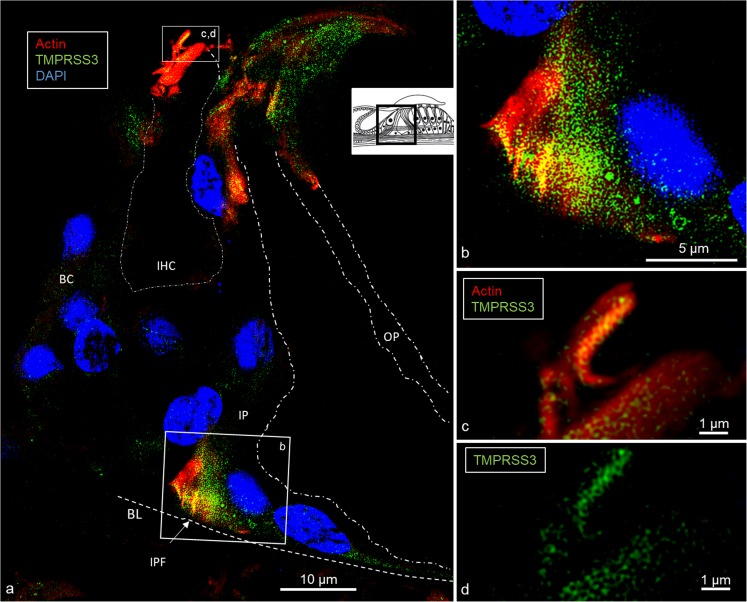
SR-SIM of actin/TMPRSS3 immunohistochemistry in the human organ of Corti. **a** Actin/TMPRSS3 immunohistochemistry showing co-staining in inner hair and pillar cells (*BC* border cell, *IPF* inner pillar foot, *IP* inner pillar cell, *OP* outer pillar cell, *dotted lines* delineate cell regions). *Framed areas* in **a** are shown at higher magnification in **b**, **d**. *Inset* in **a** Representation of organ of Corti with area of interest (*boxed*). **b** Inner pillar foot expresses both proteins. **c**, **d** Cuticula and stereocilia of IHC strongly express actin and TMPRSS3

Confocal microscopy and SR-SIM showed TMPRSS3 antibody labeling in both IHCs and OHCs at the investigated levels (Fig. 1). Parvalbumin was immunostained in the cytoplasm of both IHCs and OHCs and helped to localize the TMPRSS3 protein within hair cells (Liu et al. 2009). The proteolytic enzyme was heavily expressed in the sterocilia of both IHCs and OHCs (Figs. 2, 3) and in the cuticula in the apical portion of the cell bodies. At one level, TMPRSS3 expression in the cuticula of an IHC was surveyed in the stereocilium (lower inset in Fig. 2). TMPRSS3 decorated the cilia differently. Sometimes, actin staining of the cilium was less intense centrally with heavy TMPRSS3 staining, whereas at other times, the protein staining was more intense superficially. OHCs also expressed TMPRSS3 in vesicles located in the apical cytoplasm (Fig. 1b). These vesicles seemed to correspond to electron-dense bodies visualized by TEM (Fig. 1c, d). The TMPRSS3 antibody stained the pillar cells diffusely but revealed heavy concentrations of TMPRSS3 in the pillar head and foot. Fluorescence was quantified by using area analysis of the various regions expressing TMPRSS3 (Tables 3, 4). OHCs showed a strong parvalbumin signal in some cytoplasmic vesicles separate from TMPRSS3. The parvalbumin antibody also labeled the nerve fibers beneath OHCs and IHCs and the tunnel spiral and crossing fibers.

**Table 3:** Intensity analysis of TMPRSS3 immunoreactivity in pillar cells of human organ of Corti (mean intensity data based on four human specimens)

Cell type	Intensity		
Pillar cells	Head	Foot	Body
Outer	147	140	51
Inner	102	112	50

**Table 4:** Intensity analysis of TMPRSS3 immunoreactivity in hair cells of human organ of Corti (mean intensity data based on four human specimens)

Cell type	Intensity		
Hair cells	Stereocilia	Supra-nuclear area	Cell body
Outer	155	25	19
Inner	135	29	34

**Fig. 4:**
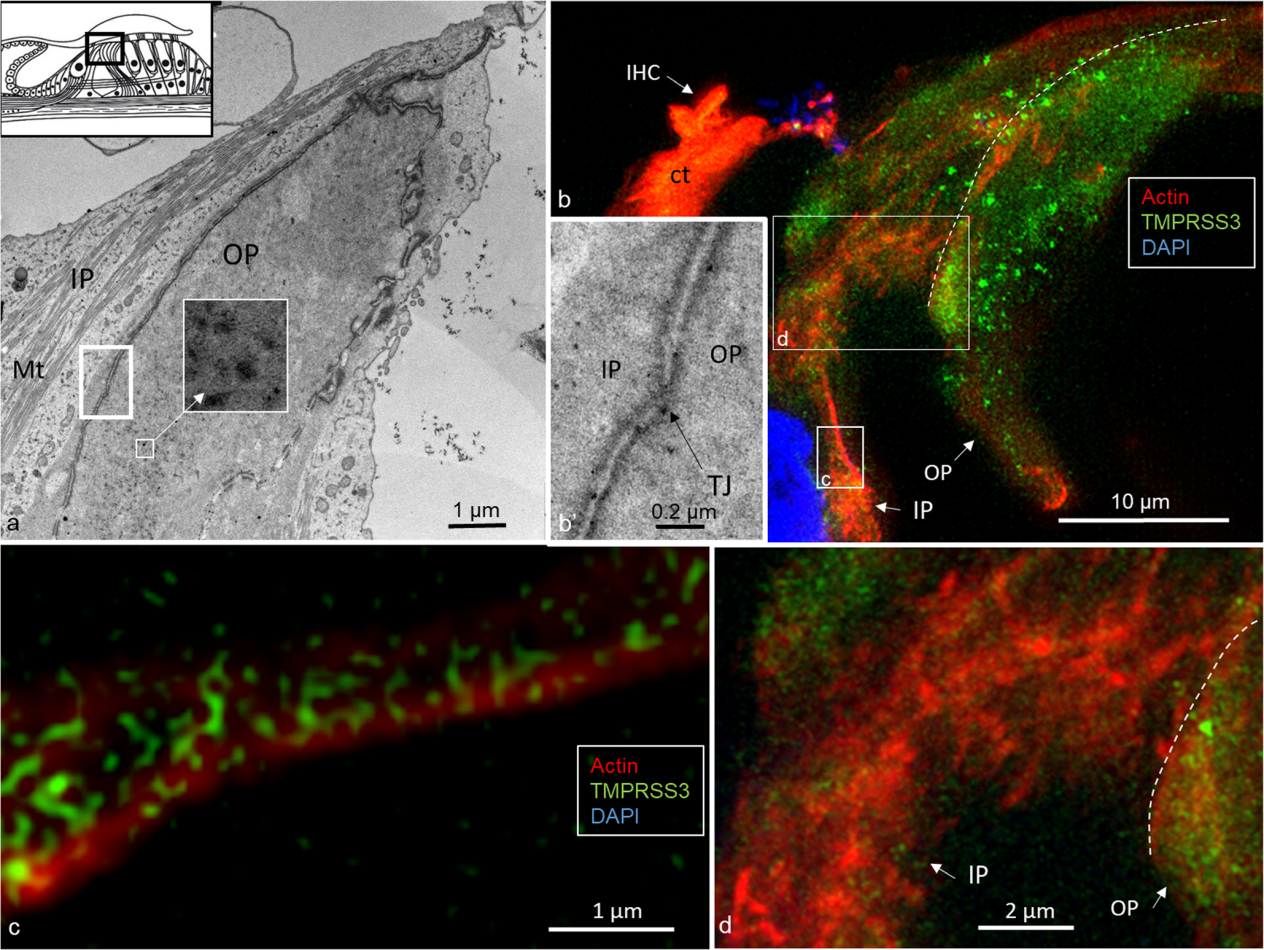
TEM and SR-SIM of the pillar head region. **a** TEM of pillar heads from a human cochlea (*IP* inner pillar cell, *OP* outer pillar cell, *Mt* microtubule). *Inset top left* Representation of organ of Corti with region of interest (*boxed*). *Left frame* is shown at higher magnification in **b’**, which reveals electron-dense precipitates (*TJ* tight junction). **b** Densities in the TEM image seem to correspond to the focal regions expressing TMPRSS3 (* IHC* inner hair cell, *Ct* cuticula, *broken line* border between heads of IP and OP cells). *Framed areas* are magnified in **c**, **d**. **c** TMPRSS3 is expressed at actin sites (single optical section). **d** Irregularly arranged actin fibrils can be seen in the head of the IP cell

**Fig. 5:**
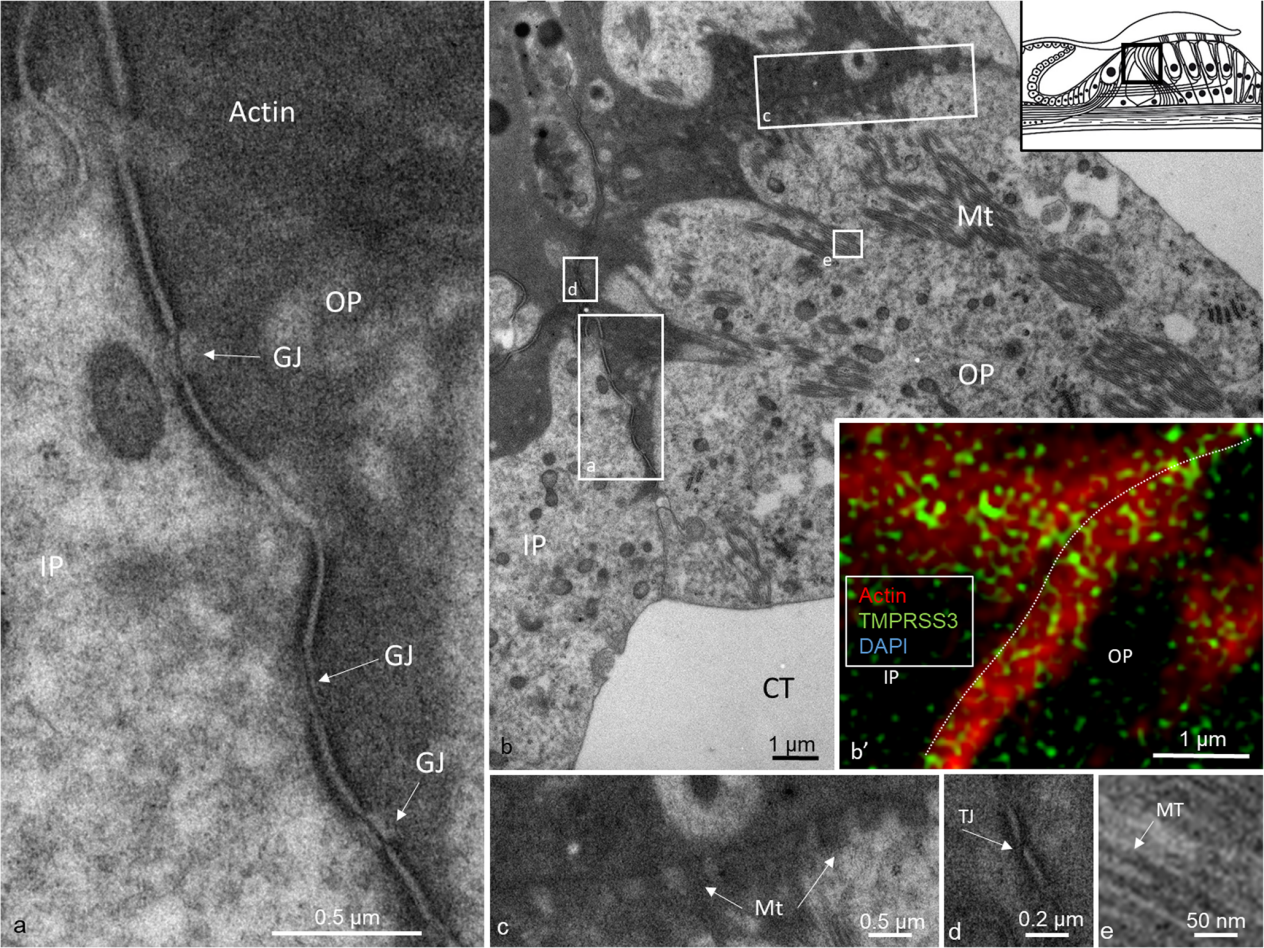
TEM of cell junctions between pillar heads. **a** Electron-dense region of a medial surfoskelosome and electron densities of adherence junctions. Several gap junctions (*GJ*) are seen between inner (*IP*) and outer (*OP*) pillar cells. **b** Low magnification imaage showing cell membrane specializations with electron-dense regions expressing actin. Microtubules can be seen to be closely related to the cell membrane. *Framed areas* are shown at higher magnification in **c-e**. *Inset top right* in **b** Representation of organ of Corti with region of interest (*boxed*). **b’** Surfoskelosome expressing actin and TMPRSS3. **c–e** *Framed areas* in **b** at higher magnification showing detail of microtubules (*MT*) in an OP cell (**c**, **e**) and a tight junction (*TJ*) between the IP and OP cells (**d**)

**Fig. 6:**
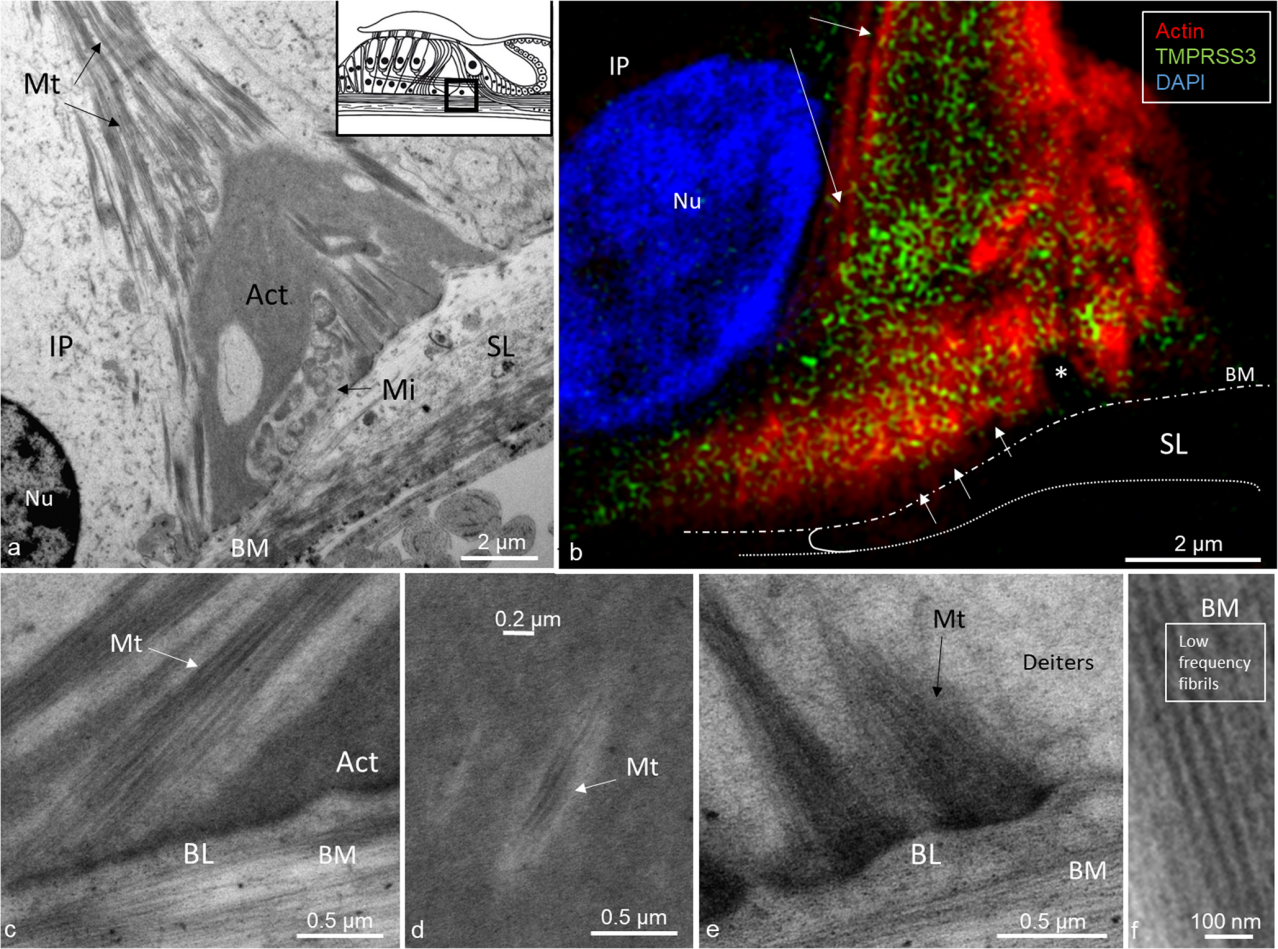
TEM and actin/TMPRSS3 immunohistochemistry of the inner pillar foot showing basal surfoskelosome and microtubule arrangement. **a** By TEM, an inner pillar cell (*IP*) is seen to rest on the lateral slope of the osseous spiral lamina (*SL*). The pillar foot faces the basal lamina and basilar membrane (*BM*). Microtubules (*Mt*) inside the cell either run independently of the surfoskelosome or are connected to the basal lamina (*Nu* nucleus, *Mi* mitochondria, *Act* actin). *Inset* in **a** Representation of organ of Corti with area of interest (*boxed*). **b** SR-SIM (maximum intensity projection) of a basal surfoskelosome expressing actin and TMPRRS3. The “empty” stripes may represent microtubules within the actin-rich electron-dense region (*upper arrows*). *Small arrows* indicate basal interruptions in actin staining near focal adhesions (inset). **c**, **d** Magnified TEM image showing detail of microtubules (*Mt*) in IP cell (*BL* basal lamina). **e** TEM of membrane specializations at the base of a Deiters cell. **f** Radial fibers of the basilar membrane

Actin was strongly expressed in association with TMPRRS3 in the stereocilia, the cuticular plates of the IHCs and OHCs and, to some degree, the reticular lamina (Figs. 1, 2, 3). TMPRRS3 and actin were expressed together in the pillar cells, especially in the head and feet regions (Figs. 3, 4, 5, 6). Co-staining showed floccular TMPRSS3 expression associated with actin but without merging or co-labeling. TMPRSS3 staining was also identified without actin. The electron-dense regions of the head and foot of the pillar cells, termed surfoskelosomes by Henderson et al. (1994), were associated with the cell membrane. These regions strongly co-expressed actin and TMPRRS3, with areas in which TMPRSS3 was solely expressed (Fig. 6). The actin fibers were regularly arranged in the pillar column (Fig. 5b, c), whereas they were arranged more irregularly in the pillar heads (Fig. 4d). Deiters cells also expressed TMPRSS3 and actin in the basal bodies. These surfoskelosomes were often smaller but were highly electron-dense. Border cells expressed TMPRSS3, but not actin. The weak staining of the type I and II spiral ganglion cells and the stria vascularis epithelium could not be determined as specific (not shown). No nerve fibers expressed TMPRSS3. The anti-K_Ca_1.1 (KCNMA1) antibody labeled hair cell bodies and cell nuclei variably and the lamina reticularis, but its specificity could not be confirmed.

TEM revealed that the surfoskelosomes were closely associated with microtubules. The electron-dense areas were present in the head and foot regions of pillar cells and at membrane regions facing the outer hair cells (phalangeal) and inner hair cells (medial) (Figs. 1, 4, 5, 6). Microtubules curved and entered these regions and faced the interior surface of the cell membranes with several focal adhesions and adhesion junctions, both basally, between the pillars heads and against hair cells (Figs. 4, 5). The inner and outer pillar head surfoskelosomes often faced each other at cell borders without any signs of intercellular specialization or bridging across the space. However, the cell membrane showed extensive adhesion junctions. Moreover, the cell membranes between the inner and outer pillar heads were decorated with prominent gap junctions, suggesting that they were electrically coupled (Fig. 5a). Surfoskelosome-independent microtubules were also observed at the pillar feet with microtubules running directly to the surface cell membrane. Some sections did not exhibit prominent basal surfoskelosomes. Supporting cells and outer hair cells contained vesicles that were of variable electron-density and that were usually located in the apical cytoplasm. These vesicles mostly had a less electron-dense center. Within the apical cytoplasm of outer hair cells, they resembled TMPRSS3-positive spherical granules (Fig. 1b, d).

## Discussion

Current attempts to regenerate cochlear sensorineural structures encourage further examination of the molecular expression and microstructure of human cochlear hair and supporting cells along the cochlear axis. In addition, an exploration of the mechano-electric transduction channels present in hair cell stereocilia and their maintenance is under way (Ueyama et al. 2014; McGrath et al. 2017). Because of difficulties in collecting fresh tissue, few studies of the human cochlea have been undertaken involving the use of high-resolution fluorescence microscopy. The present investigation is the first to identify proteins and ion channels related to human hair cell transduction and cell motility by using high-resolution immunofluorescence microscopy techniques. Application of such analysis tools was successful in recent studies of the human cochlea (Liu et al. 2016, 2017a, 2017b). The limited number of tissue specimens and available sections are major disadvantages. Rare samples here have been collected, after ethical permission, from tissue sacrificed for the complete and safe removal of life-threatening petro-clival meningioma.

### TMPRSS3 and actin in hair cells

Our study has shown that the TMPRSS3 protein is associated with actin in the human organ of Corti. These findings suggest that this proteolytic enzyme plays a major role in the action of the prominent actin fiber networks. TMPRSS3 is strongly expressed in IHC and OHC stereocilia and is detectable from the cuticula to the uppermost area of the stereocilium. In the supra-nuclear region of the OHCs, vesicles express TMPRSS3 and may represent storage organelles of the produced enzyme involved in actin-related signal processing. Similar-sized electron-dense vesicles have been found in TEM images and are also seen in border and Deiters cell phalanxes. Both IHCs and OHCs strongly express the Ca^2+^ buffer parvalbumin. A high Ca^2+^ buffering capacity may be critical to limit Ca^2+^ diffusion for the proper function of transduction channels. The stores have also been localized in separate vesicles in the OHC cytoplasm. Hensen bodies, earlier described by Lim (1986), may represent Ca^2+^ storage depots in the subcuticular region of human OHCs (Mammano et al. 1999). The associated endoplasmic reticulum may also be involved in the secretion of the TMPRSS3 enzyme.

### TMPRSS3 and K_ca_1.1 channel expression

TMPRSS3 has been shown to regulate the outward-rectifying potassium currents in IHCs via functional K_ca_1.1 channels (Molina et al. 2013). TMPRSS3 contains low-density lipoprotein receptor class A and scavenger receptor cysteine-rich domains. It is located in endoplasmic reticulum membranes and is transferred to the plasma membrane to function through an interaction with ApoA1 (Guipponi et al. 2008). Fasquelle et al. (2011) have described complete hair cell degeneration after maturation in a mutant mouse carrying a protein-truncating nonsense mutation in TMPRSS3 (Y260X); since both IHCs and OHCs degenerate, the proteolytic activity of TMPRSS3 is concluded to be present in both cell types. A likely substrate for TMPRSS3 is K_ca_1.1, because the loss of TMPRSS3 function decreases K_ca_1.1 channel expression at the plasma membrane in mutants (Molina et al. 2013). This has been confirmed by immunohistochemistry with the down-regulation of the cochlear K_ca_1.1-associated protein APOA1. Our study has been unable to verify with certainty that K_Ca_1.1 channels are the TMPRSS3 substrate related to actin activity.

### TMPRSS3 and cell contractility in human organ of Corti

Flock et al. (1982) used cryo-ultramicrotomy and immunohistochemical methods to study contractile proteins in supporting cells in guinea pigs. They showed densely packed actin in the apex and base of cells with chains of mixed microtubules and F-actin acting as bridging struts from the base to apex. Angelborg and Engström (1972) demonstrated, via electron microscopy, the microtubule/microfilament organization in guinea pig pillar cells giving structural support to the organ of Corti. Microtubules are anchored into the cell membrane with “cement-like” substances. These structures have been described by Henderson et al. (1994) at the base, middle, and apical membrane regions and termed surfoskelosomes. They contain β- and γ-non-muscle actin isoforms and α-actinin (Slepecky and Savage 1994; Drenckhahn et al. 1985), whereas the cuticular plates contain actin, α-actinin, myosin and tropomyosin, and profilin and fodrin (Slepecky and Ulfendahl 1992; Ylikoski et al. 1992). We have found similar bodies associated with microtubules facing the basal lamina and hair cells with focal densities suggesting increased adherence. TMPRSS3 is richly expressed in the basal surfoskelosomes, particularly at the center and regions facing the cell membrane, representing encroaching microtubules. A similar arrangement has been found in the pillar head regions. These findings suggest that TMPRSS3 protein is functionally linked to the microtubule-actin network. Actin filaments and microtubules can crosslink directly or indirectly via protein complexes or signaling molecules. Such interactions with motor protein complexes are thought to be dynamic, in which microtubule-associated proteins (MAPs) that belong to the MAPs1, 2, and 4 families and Tau proteins are known directly to crosslink the two cytoskeletons in neurons (Mohan and John 2015). Slepecky and Ulfendahl (1992) have found only MAP-2 in OHCs in the organ of Corti. MAP-2 may establish filamentous processes of actin/microtubule-connecting proteins from microtubules and interact with the actin filaments (Griffith and Pollard 1978; Sattilaro et al. 1981). The architecture provides possible support for this view, suggesting that human pillar cells not only form a solid foundation for the hearing organ, but also have the capacity for active motion. As recently shown by Liu et al. (2017a), a large number of gap junctions expressing connexin-30 are present between the inner and outer pillar cells. This suggests that the cells are electrically coupled, a feature that might serve to optimize coordinated cell action in the organ of Corti (Fig. 5a).

According to Schick et al. (2003), vasodilator-stimulated phosphoprotein (VASP), a member of the ENA/VASP-protein family, is believed to regulate actin dynamics, such as motility and cell adhesion, together with zyxin. Zyxin is particularly concentrated at sites at which VASP-dependent actin dynamics occur. These proteins co-localized with the pan-actin antibody, a finding that leads the authors to believe that actin-based dynamics in the pillar cells are involved in controlling the longer-lasting mechanical properties of the cochlea.

Pillar cells undergo spectacular structural changes with microtubule bending and tunnel formation during development. These changes accomplished by contractile mechanisms within the microtubule bundles are of potential importance for active vibratory responses during hearing (Tucker et al. 1993). Slepecky et al. (1995) have shown that sensory and supporting cells in guinea pig and gerbil cochlea contain different populations of microtubules. Microtubules in sensory cells are thought to be dynamic structures, whereas those in supporting cells are stable and long-lived. This differs from the vestibular system, in which both sensory and supporting cells contain microtubules composed of tyrosinated and acetylated (but not polyglutamylated) tubulin (Ogata and Slepecky 1995). Post-translational modifications during development may be related to the differentiation into sensory and supporting cells during cell repair following trauma (Slepecky et al. 1995) and even regeneration. According to Henderson et al. (1994), mature pillar cells are probably not replaced from precursor cells. Instead, microtubules might be maintained and replaced throughout life through the microtubule-nucleating site of the centrosome. Renauld et al. (2015) have investigated the localization of β-tubulin isotypes within the hearing organ during rat development by using confocal microscopy. Except for the β3-tubulin isoform, all β-tubulin isoforms are present in supporting cells, with the β5-tubulin isoform appearing only at a key stage of post-natal development in pillar cells and Deiters cells. The results suggest that certain tubulin isoforms are involved in the neo-generation of microtubule bundles. TMPRSS3 is expressed in both sensory and supporting cells, and further characterization of the tubulin isoforms is under way.

According to Jensen-Smith et al. (2003), different cell types synthesize different subsets of isotypes in adults. IHCs display β1 and β2 tubulin, whereas OHCs display β1 and β4 tubulin. Only β2 and β4 tubulin are found in the inner and outer pillar cells, whereas β1, β2, and β4 tubulin are present in Deiters cells. Further molecular analysis of the anchoring elements in focal adhesions at the basilar membrane, including TMPRSS3, are warranted. Microtubules have a thickness of 25 nm but seem to be anchored through filamentous connections at focal adhesions. Notably, microtubules in the pillar foot also run independently of the basal surfoskelosome. Such an arrangement might have significance for the contraction/relaxation of the polarized network during oscillatory vibrations. Recently, microtubules forming load-bearing spring elements and running in parallel with the contractile apparatus have been described in the heart and are thought to regulate myocyte stiffness and contractility, influencing the resistance to contraction (Robison et al. 2016).

In brief, TMPRSS3 seems to exert several functions in the human inner ear, such as in actin-related hair cell mechanics and the support of cell contractility. The dynamic components of supporting cells may modulate mechanical vibrations generated from the basilar membrane via the microtubule-actin system along the cellular shafts to the reticular lamina and hair cells. Additional knowledge of the physiological functions of this protease and its domains is necessary in order to obtain a more complete picture of its roles in human hearing.

## Electronic supplementary material

Supplementary Figure 1 Fluorescence intensity was quantified by area analysis of TMPRSS3 immunoreactivity in the organ of Corti by using NIS Element BR-3.2. (GIF 950 kb)

High resolution image (TIFF 831 kb)
