# Supplementary figures and images for: Expression of trans-membrane serine protease 3 (TMPRSS3) in the human organ of Corti
Source: Cell Tissue Res. 2018 Feb 19;372(3):445–56. doi: 10.1007/s00441-018-2793-2 (PMC5949142; doi:10.1007/s00441-018-2793-2)

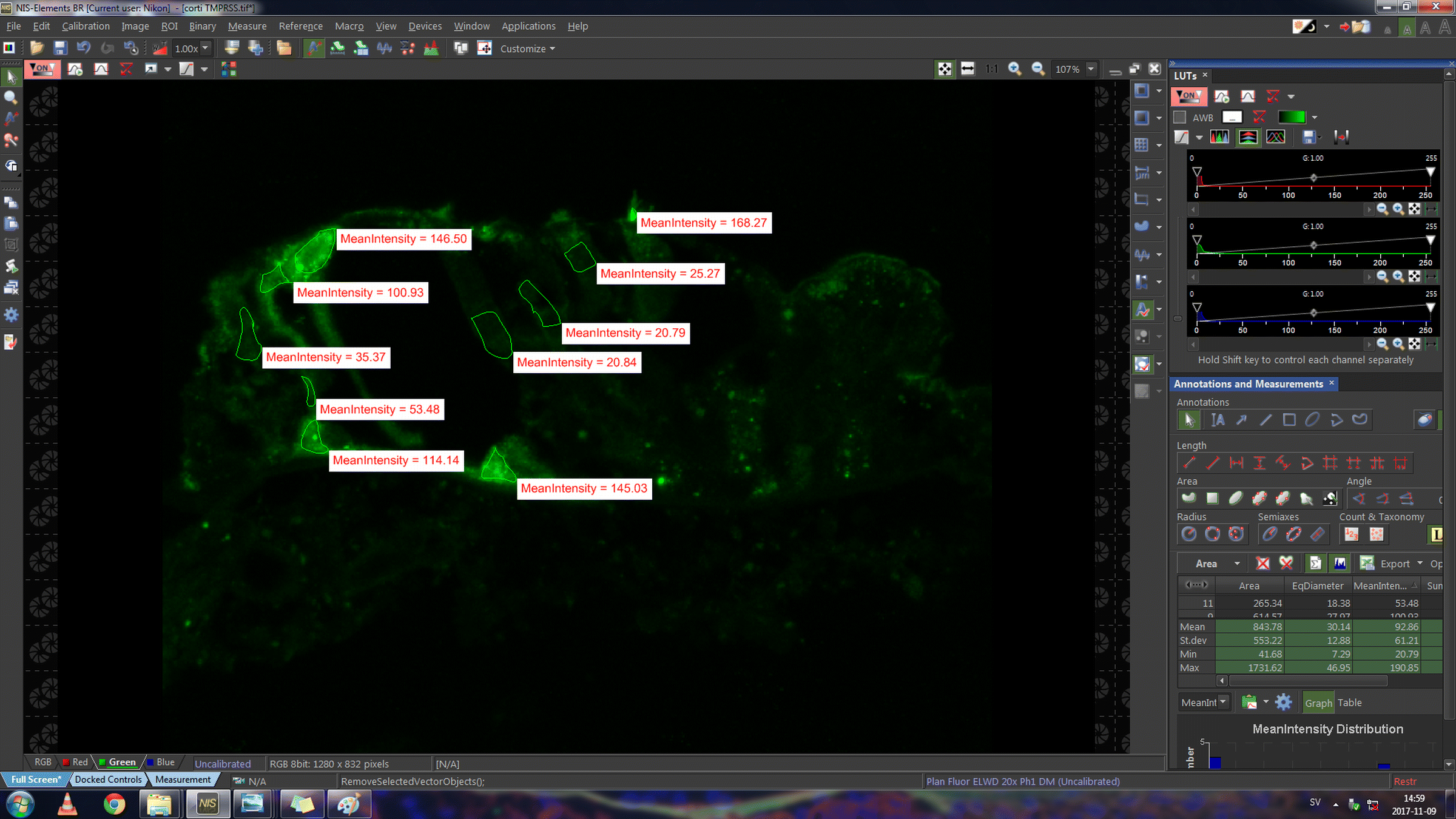

Supplement: Supplementary file 1 — Fluorescence intensity was quantified by area analysis of TMPRSS3 immunoreactivity in the organ of Corti by using NIS Element BR-3.2. (GIF 950 kb) [file 441_2018_2793_Fig7_ESM.gif]

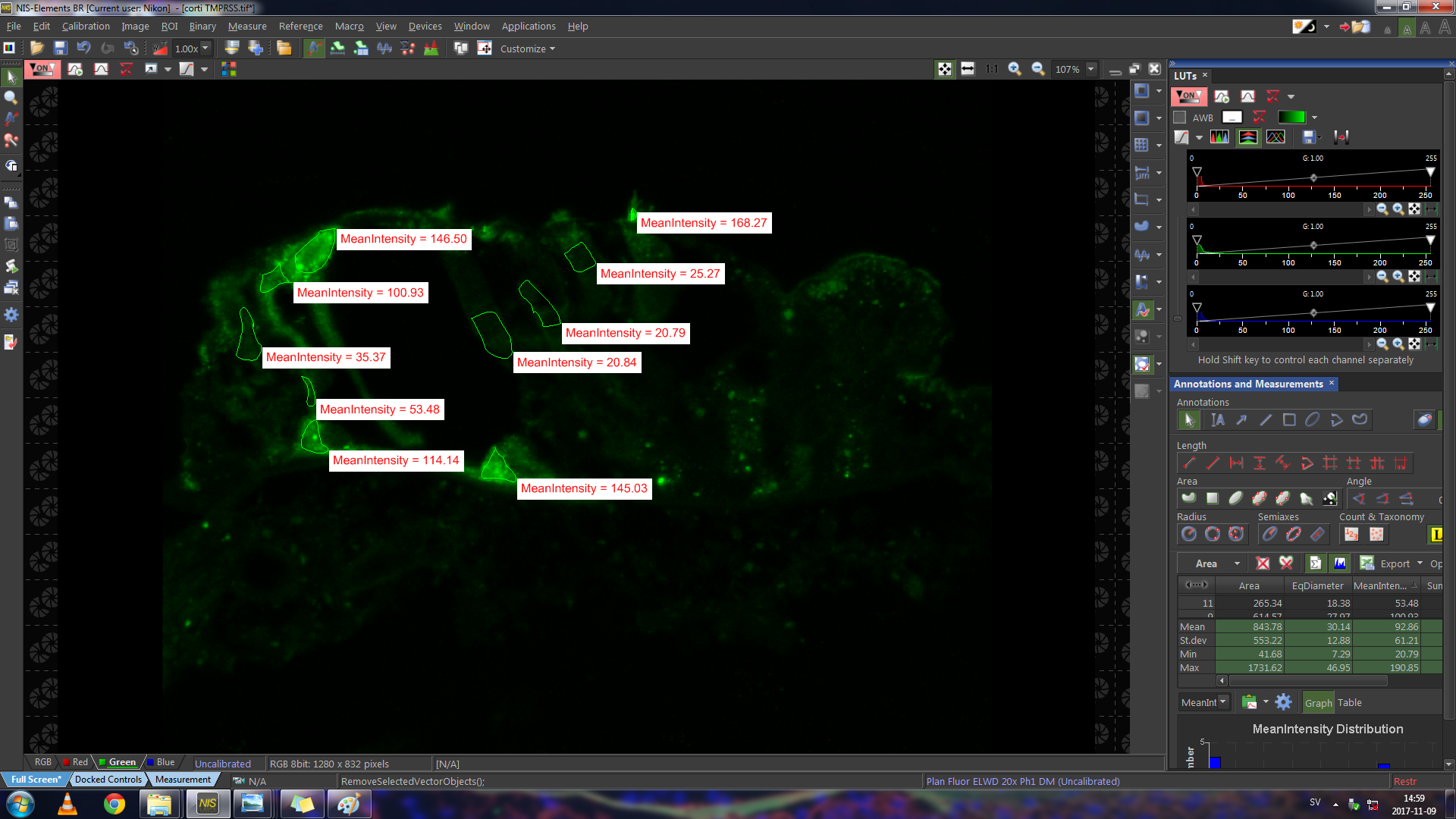

Supplement: Supplementary file 2 — High resolution image (TIFF 831 kb) [file 441_2018_2793_MOESM1_ESM.tif]
